# The measurement method matters

**DOI:** 10.1107/S2052252526004434

**Published:** 2026-04-30

**Authors:** Holger Klein

**Affiliations:** a Université Grenoble Alpes and Centre National de la Recherche Scientifique 25 rue des Martyrs – BP 166 38042Grenoble France

**Keywords:** 3D ED, electron diffraction, structure determination, benchmarking

## Abstract

The systematic comparison of 3D-ED methods in terms of data quality presented by Schmitt *et al.* [*IUCrJ* (2026), **13**, 291–303] is discussed.

In the long quest for determining the structure of materials in order to better understand their properties, great progress has been made in recent years by the development of several electron diffraction methods. These methods are complementary to the traditional X-ray and neutron diffraction techniques, since electron diffraction can be used to obtain single-crystal diffraction data from crystals as small as a few tens of nanometres in diameter, *i.e.* from volumes a million times smaller than for X-rays. This is an important advantage whenever growing micron-sized single crystals of sufficient quality is tedious, time-consuming or altogether impossible.

These techniques have been grouped under the name of 3D-ED (Gemmi *et al.*, 2019 ▸) and yield data qualities comparable to X-ray and neutron diffraction, allowing researchers not only to establish preliminary structural models of unknown crystalline phases but also to refine those models in order to obtain information about structural details such as charge density, absolute configuration, non-sphericity of atoms and so on.

The common point of the 3D-ED techniques is that they all use electron diffraction in a transmission electron microscope (TEM) or in a dedicated electron diffractometer in order to obtain three-dimensional structural information. However, the data acquisition strategies show differences that might have an impact on the data quality and therefore on the precision that can be obtained when determining structural details.

In this issue of *IUCrJ*, Schmitt *et al.* (2026 ▸) report on a systematic study of the influence of the data acquisition strategy on data quality. They mainly compare three different data acquisition strategies: stepwise static rotation, precession electron diffraction tomography (PEDT) (Mugnaioli *et al.*, 2009 ▸) and continuous-rotation electron diffraction (cRED) (Nederlof *et al.*, 2013 ▸). The latter two are nowadays the most frequently used 3D-ED data acquisition techniques for structure determination (Klar *et al.*, 2023 ▸).

In stepwise static rotation the crystal is rotated by discrete angular steps around an arbitrary axis and diffraction data are recorded at each step while the crystal is static (Fig. 1 ▸). This leads to a discrete sampling of the reciprocal space, potentially missing important information in between successive steps. In PEDT, a similar stepwise rotation of the crystal is done, but at each position the crystal is subjected to an incident beam that is precessed around the optical axis of the TEM, with the effect that the entire reciprocal space is scanned. In cRED, as the name suggests, the crystal is rotated in a continuous manner while a digital camera records the diffraction with a predefined frame rate. Here also, the entire reciprocal space is sampled except for wedges during the read-out time of the camera. For cRED, the authors point out that there are several possible pitfalls that might diminish the data quality. Introducing new software, they show how these pitfalls can be avoided, resulting in higher quality data.

All of these techniques have been successfully applied to solve crystal structures, but up to now there has been no clear evidence as to which of these strategies yields the best quality data, allowing the subtlest structural details to be determined. This is exactly the question that is addressed in the article by Schmitt *et al.*

For this goal the authors chose three ceramic compounds. Two of these contained low-occupancy, mobile sodium and lithium ions, which contribute only little to the diffraction intensities, so their detection and quantitative determination depend critically on the diffraction data quality.

In order to explore only the influence of the measurement strategy on the data quality, the authors took care to eliminate other possible influences. To eliminate effects stemming from different intrinsic qualities of different crystals, such as crystallinity, mosaicity and thickness, the three measurement strategies were applied to the same crystals successively. In addition, the illumination conditions in the TEM and the tilting range for the crystals were set similarly. From the datasets that were obtained the structures were solved and refined taking dynamical diffraction effects into account.

There are different ways of assessing the quality of a structural model of a crystal obtained from diffraction data, and these are more or less sensitive to small differences in data quality. The direct results of the refinement, like the crystallographic *R* factor, refinement statistics or the deviation of atom positions from their known positions, can be analyzed. Calculating difference electrostatic potential (DESP) maps and their residual noise allows a deeper analysis of the quality, as does consideration of the statistics for the individual reflection intensities. The authors apply and compare all three.

Schmitt *et al.* clearly identify stepwise static diffraction as yielding less good structure models than cRED and PEDT. For the latter two the authors obtain similar precision on the atomic positions. In terms of refinement statistics, however, the values obtained by PEDT were better, in one case even achieving single-crystal X-ray diffraction quality.

By considering the measured intensities, the authors show that the high-intensity reflections are under-estimated compared with the calculated intensities, as would be expected for dynamical diffraction effects, but this persists in the data even though the refinement procedure takes dynamical effects into account. The effect is less pronounced for PEDT data than for cRED data, indicating a higher quality in this case. This is also seen in the DESP maps, where the residual noise in the maps is lower for PEDT.

In summary, Schmitt *et al.* contribute here an important study on the quality of diffraction data as a function of the measurement strategy. They show that the data quality achievable by cRED and PEDT is sufficiently high to identify weak scatterers with only partial occupancy within ceramic materials. These methods can be applied to nanometre-sized crystals, much too small for single-crystal X-ray diffraction, opening new research fields. The authors have demonstrated this on solid-state electrolytes, but in many other fields of materials sciences, from intermetallics to coordination polymers, their results can pave the way for new structural studies.

## Figures and Tables

**Figure 1:**
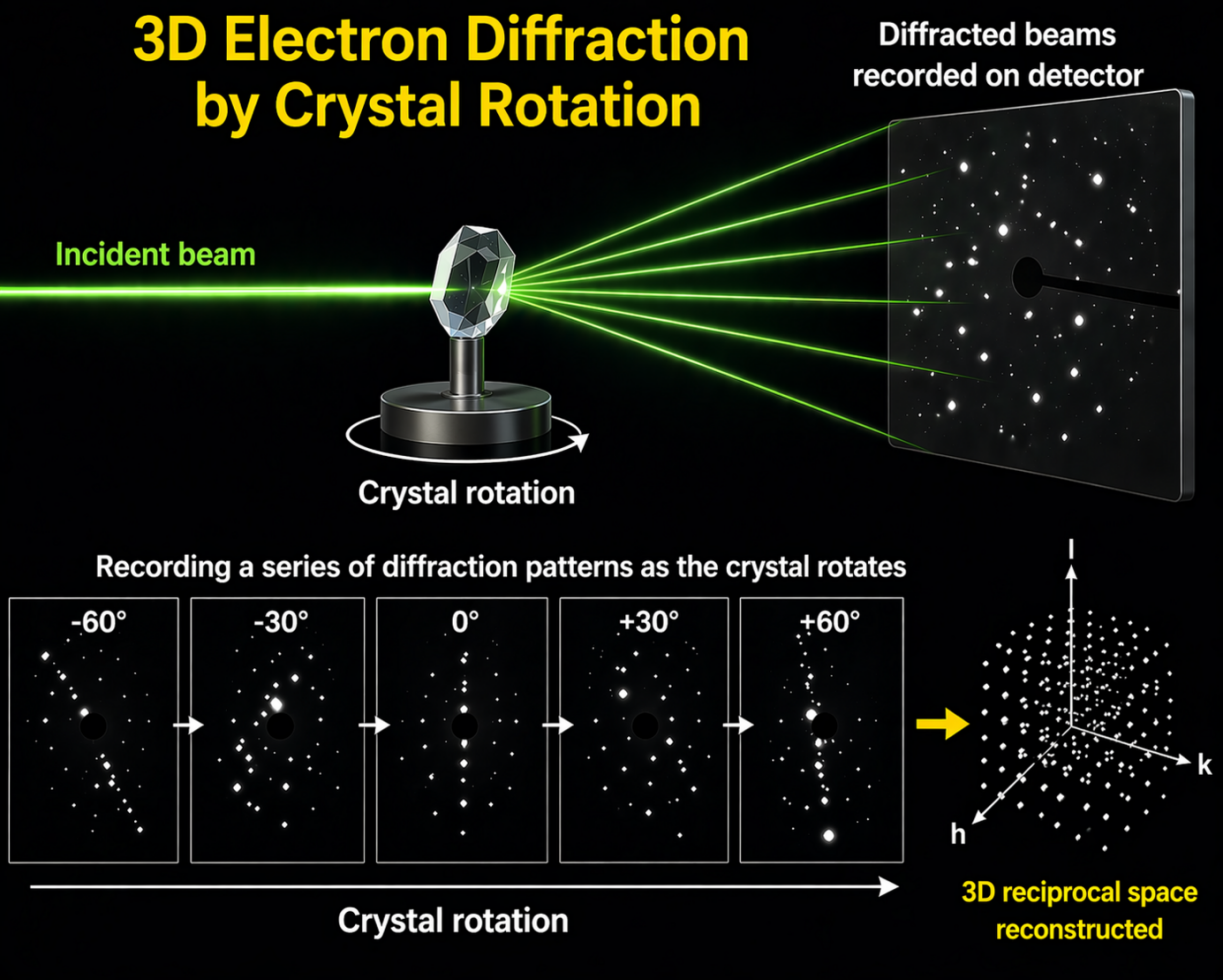
Illustration of a 3D-ED experiment where diffraction patterns are taken during the rotation of crystal in order to reconstruct the 3D reciprocal space. The image was generated with the help of *ChatGPT* by OpenAI.
